# Primary amelanotic malignant melanoma of the small intestine diagnosed by esophagogastroduodenoscopy before surgical resection

**DOI:** 10.1007/s12328-013-0380-3

**Published:** 2013-04-13

**Authors:** Takanori Suganuma, Junko Fujisaki, Toshiaki Hirasawa, Akiyosi Ishiyama, Yorimasa Yamamoto, Tomohiro Tsuchida, Masahiro Igarashi

**Affiliations:** Department of Gastroenterology, Cancer Institute Ariake Hospital, 3-8-31, Ariake, Koto-ku, Tokyo 135-8550 Japan

**Keywords:** AMM, Melanoma, DAV, Small intestine

## Abstract

A 67-year-old man, presenting with anemia and suspected gastric cancer, was referred to our hospital, where he underwent esophagogastroduodenoscopy (EGD). Biopsy revealed densely populated semi-circular cells with abundant cytoplasm that were positive for S-100 protein, melanoma antigen, and HMB-45, resulting in a diagnosis of malignant melanoma. A gastrointestinal barium study for further exploration demonstrated a filling defect 6 cm in size at the ligament of Treitz. Follow-up EGD of this finding revealed an ulcerated, half-circumferential lesion with a distinct ulcer mound extending from the ascending part of the duodenum to the jejunum, and additional biopsy also indicated malignant melanoma. Computed tomography scans showed wall thickening from the ascending part of duodenum to the proximal jejunum, whereas positron emission tomography revealed accumulation at the upper gastric body, the duodenum to the jejunum, and the left adrenal gland. Systemic exploration of the patient, including the skin, anus, and eyeballs, revealed no other lesions, and primary small intestinal malignant melanoma with metastasis to the stomach and adrenal gland was diagnosed. Partial duodenojejunectomy, partial gastrectomy, and left adrenalectomy were performed, and adjuvant chemotherapy with dacarbazine, nimustine hydrochloride, and vincristine sulfate was administered. No postoperative recurrence has been observed in the past 3 years.

## Introduction

Malignant melanoma develops primarily in the skin, the transitional zone between the skin and mucosa, and the eyeballs. Primary gastrointestinal malignant melanoma (PGIM) is rare (occurring in approximately 1.0 % of cases) [1], progresses quickly, and most often occurs in the anus, rectum, and esophagus. PGIM rarely occurs in the small intestine, and a diagnosis is usually determined by postoperative resected specimen pathology. Here, we report a case of primary amelanotic malignant melanoma (AMM), which developed in the small intestine and was identified by preoperative tissue biopsy.

### Case report

**Fig. 1:**
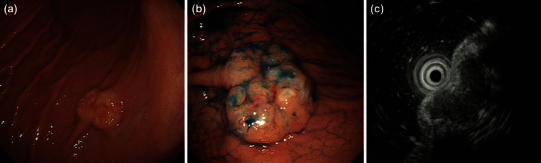
Esophagogastroduodenoscopy. **a** A 2-cm SMT-like elevated lesion with a depression was observed at the posterior wall of the middle gastric body. **b** Indigo carmine chromoendoscopy: the surface of the tumor was lobular. **c** EUS revealed a low echoic mass located at the first to third layers

**Fig. 2:**
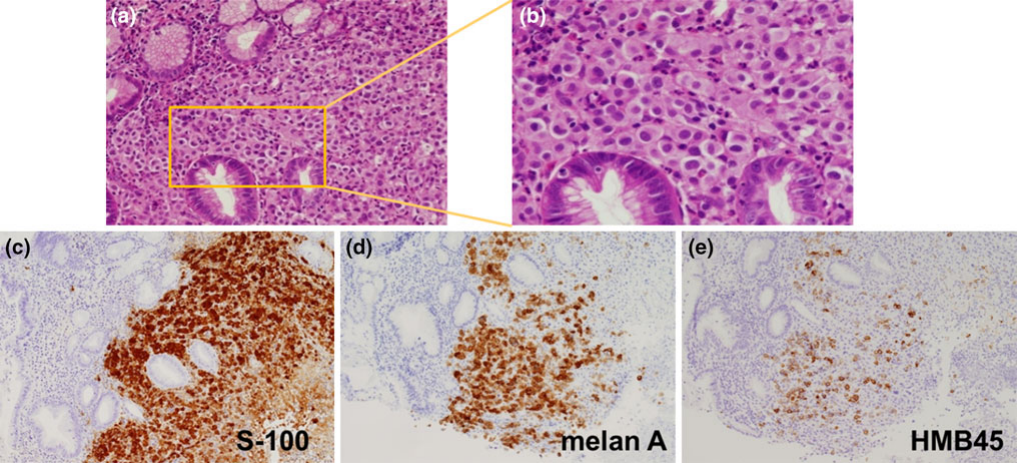
**a** Low-power field and **b** high-power field: H&E staining of the biopsy specimens from the gastric tumor. A dense population of semi-circular cells was observed. Pigmented granules were not observed. Immunohistochemistry: **c** positivity for S-100 protein; **d** positivity for Melan A; and **e** weak positivity for HMB-45

A 67-year-old man, who first felt dyspnea upon exercise and fatigue in October, 2006, was found to have anemia (hemoglobin 5.6 g/dl) in April 2007 and was referred to our hospital on May 8, 2006. Patient characteristics and initial laboratory results are presented in Table 1. Esophagogastroduodenoscopy (EGD) was performed, and a 2-cm submucosal tumor-like elevated lesion with a depression was observed at the posterior wall of the middle gastric body. The adjacent mucosa showed no abnormality, and endoscopic ultrasonography showed a low echoic mass with a distinct margin located in the second to third layer (Fig. 1a, b, c). Biopsy of the gastric tumor determined densely populated semi-circular cells, which were positive by immunohistochemical staining for S-100 protein and melanoma antigen (Melan A) and weakly positive for HMB-45 (Fig. 2a, b, c, d, e).

**Table 1:** Laboratory results at the first visit. Iron deficiency anemia (Hb 7.6 g/dl; MCV 70.7; and Fe 26 μg/dl) was observed, but biochemistry and tumors markers showed no abnormality

Peripheral blood		Chemistry			
WBC	6400/µl	TP	7.0 mg/dl	Ca	8.8 mEq/l
RBC	379 × 10^4^/µl	ALB	4.0 mg/dl	CRP	0.1 mg/dl
Hb	7.6 g/dl	T-Bil	0.3 mg/dl	Glu	93 md/dl
MCV	70.7	AST	18 IU/l	Ferritin	6.9 ng/ml
MCH	20.1	ALT	30 IU/l	Fe	26 µ/dl
PLT	53.5 × 10^4^/µl	LDH	139 IU/l	Serology	
Coagulation		γGTP	16 IU/l	HBsAg	(−)
PT/INR	1.09	BUN	19.0 mg/dl	HCVAb	(−)
APTT	33.3 s	Cr	0.85 mg/dl	CEA	1.0 ng/ml
		Na	141 mEq/l	CA19-9	2.0 ng/dl
		K	4.2 mEq/l	AFP	3.7 ng/ml
		Cl	103 mEq/l

**Fig. 3:**
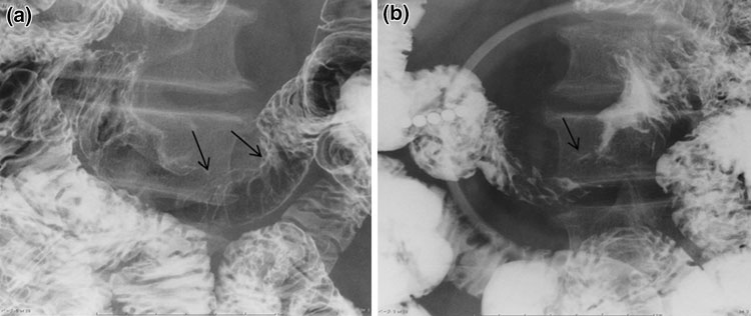
**a**, **b** A barium study revealed a filling defect 6 cm in size at the site close to the Treitz ligament (*black arrow* in **a**, **b**)

**Fig. 4:**
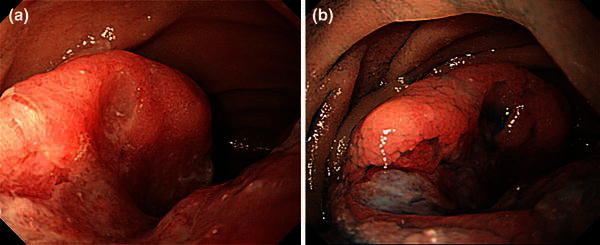
**a** Esophagogastroduodenoscopy for the second time showed a type 2-like tumor half circumferential at the ascending part of duodenum to the jejunum. **b** Indigo carmine chromoendoscopy: dye spraying visualized the ulcer margin more clearly

**Fig. 5:**
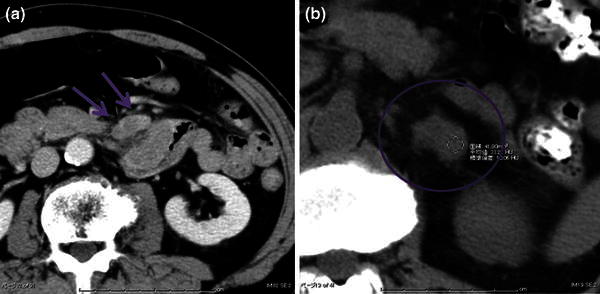
**a** Wall thickening was observed from the ascending part of duodenum to the proximal jejunum (*blue arrow* in **a**). **b** A lobular tumor was detected at the lateral side of the left adrenal (*blue circle* in **b**)

**Fig. 6:**
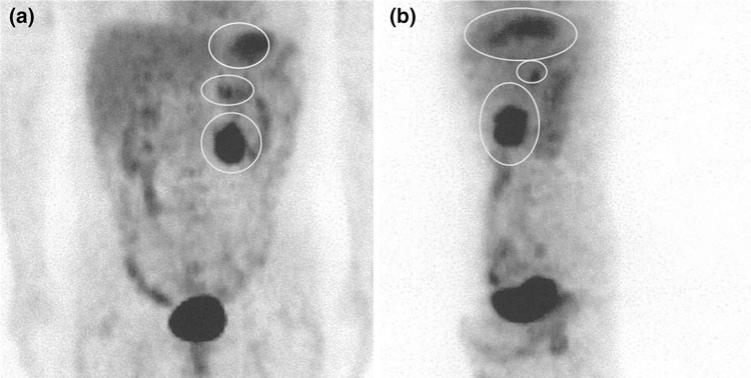
**a** Frontal view, **b** lateral view: PET showed accumulations at the upper gastric body, duodenum and left adrenal (*white circle* in **a**, **b**)

A gastrointestinal barium study was performed for further exploration of the gastric lesion that showed a filling defect 6 cm in size adjacent to the ligament of Treitz (Fig. 3a, b). Follow-up EGD was performed and the intestinal lesion was determined to be an ulcerated, half-circumferential lesion with a distinct ulcer mound extending from the ascending part of the duodenum to the jejunum. Biopsy of the intestinal lesion indicated melanoma, as previously identified in the stomach (Fig. 4a, b). Wall thickening was observed by computed tomography scanning from the ascending part of duodenum to the proximal jejunum, and a 2-cm lobular tumor was observed at the lateral surface of the left adrenal gland (Fig. 5a, b). Positron emission tomography showed accumulation in the upper gastric body, the duodenum, and the left adrenal gland (Fig. 6a, b).

**Fig. 7:**
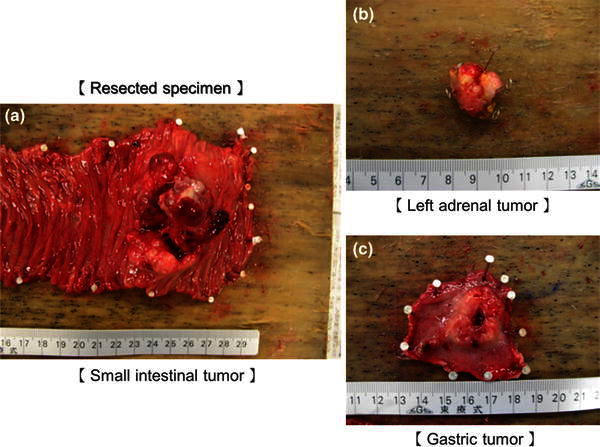
**a** Diameter of the small intestinal tumor, 62 × 46 mm; **b** diameter of the left adrenal tumor, 20 × 15 mm; and **c** diameter of the gastric tumor, 27 × 22 mm

**Fig. 8:**
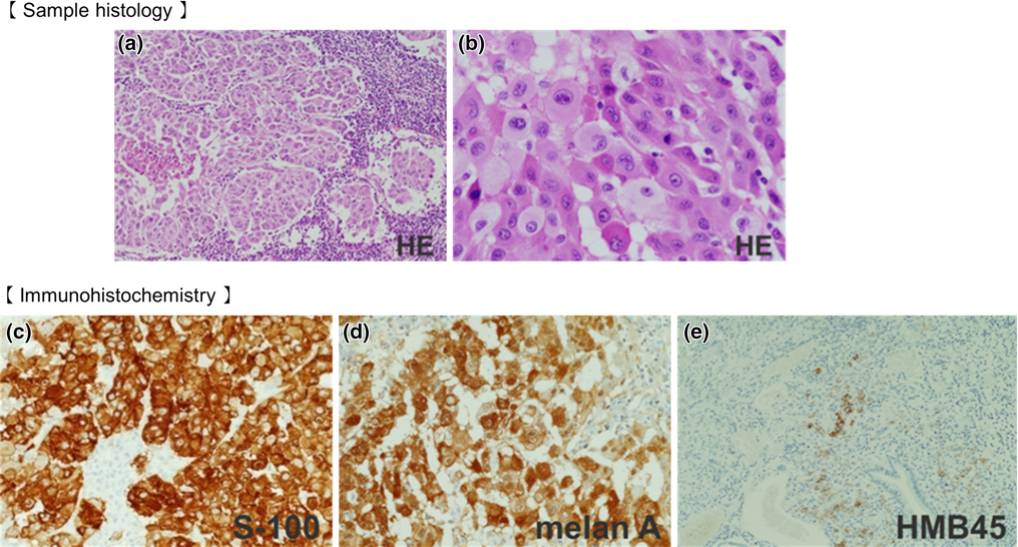
H&E staining revealed stranded proliferation of spindle-shaped cells and higher magnification views showed bright cytoplasm and nuclear atypia (**a** lower magnification; **b** higher magnification). Immunohistochemistry: **c** negativity for HMB-45; **d** positivity for S-100 protein; and **e** positivity for Melan A. Deposition of brownish black pigmented granules, usually recognized in melanotic melanoma, was not observed

Systemic exploration was performed, and the patient’s body surface and eyeballs were examined, with no abnormal findings observed. These results suggested that the primary lesion was in the small intestine, and partial duodenojejunectomy, partial gastrectomy, and left adrenalectomy were performed. The identified small intestinal tumor was 62 × 46 mm in size and had no recognized lymphatic metastasis. In the resected specimens, blackish changes were not observed (Fig. 7a, b, c). Hematoxylin and eosin (H&E) staining revealed stranded proliferation of spindle-shaped tumor cells, and bright cytoplasm and nuclear atypia were recognized by high power magnification field observation. Deposition of pigmented granules, which would be typical in melanotic melanoma, was not observed. Immunohistochemical staining revealed that the primary intestinal tumor was negative for HMB-45 but positive for S-100 protein and Melan A, which led to the diagnosis of AMM (Fig. 8a, b, c, d, e). Given the high risk for recurrence of the disease, adjuvant chemotherapy with dacarbazine (DTIC), nimustine hydrochloride, and vincristine sulfate (DAV) was administered for 5 cycles, and the patient has been recurrence free for 3 years.

## Discussion

Malignant melanoma occurs at an approximate rate of 10 per 100,000 in Western countries, but is a markedly rare malignant tumor in Japan, occurring at a rate of 1–1.5 per 100,000. In Japan, approximately 80 % of diagnosed malignant melanomas occur in their primary form on the skin (32.7 % in the transitional zone between the skin and mucosa, 27.1 % in the nasal and oral cavities, and 21.4 % in the eyeballs). This is because neuroectodermal melanocytes are abundantly present in the skin; the mucosal epithelium of the nasopharynx, oral cavity, and rectum; the cranial pia mater; and the choroid of the eyeballs. It is extremely rare for malignant melanomas to occur at other primary sites in the body [1]. A study on malignant melanomas originating from the gastrointestinal tract showed that the primary sites of the cancer were the oropharynx and nasopharynx (32.8 %), anal canal (31.4 %), rectum (22.2 %), esophagus (5.9 %), stomach (2.7 %), small intestine (2.3 %), gallbladder (1.4 %), and colon (0.9 %) [2].

In the present case, malignant melanoma was observed in the small intestine, stomach, and left adrenal gland, with the largest tumor detected in the small intestine. A diagnosis of malignant melanoma originating in the small intestine was confirmed because the largest lesion was in the small intestine; no changes were seen in the skin, eyeballs, esophagus, rectum, and transitional zone from the rectum to the anus, and no lymphatic metastasis was seen in the small intestinal lymph nodes either. In this case, the metastases to the stomach and adrenal gland were thought to occur through local hematogenous metastasis and not by direct invasion. The direct invasion for other organs was absent in the resected specimen. The small intestinal lesion was confirmed in the second EGD, and an iteration biopsy was necessary for differentiation with other disease. There has long been a concern that biopsy of primary melanoma could lead to an increase rate of micrometastases and an increased risk of recurrence. However, in a report of cutaneous malignant melanoma, there was no significant difference found between the 5-year overall survival (OS) rate, local recurrence rate, rate of melanoma-associated death, and metastasis rate for sentinel lymph nodes between a total resection biopsy group and a partial biopsy group [3–6].

AMM is a rare disease that accounts for approximately 2 % of all malignant melanomas and is often diagnosed during the follow-up and treatment of a different disease. While the 5-year OS rate of malignant melanoma is 50–60 %, AMM has an extremely poor prognosis, with a 5-year OS rate as low as 25 % [7, 8]. This poor prognosis may be due to delayed diagnosis due to macroscopic problems and abnormal melanin synthesis in addition to undifferentiated tumor characteristics [6]. In AMM, melanin granules cannot be confirmed by H&E staining or may be present at extremely low levels. Macroscopically, the tumor is white or grayish white and is difficult to differentiate from malignant tumors such as undifferentiated cancer and gastrointestinal stromal tumor [9, 10]. For definitive diagnosis, immunohistochemical staining using anti-S-100, anti-Melan A, and anti-HMB-45 antibodies is indispensable. In the present case, the gastric lesion was weakly positive for HMB-45, while the primary lesion in the small intestine was negative for HMB-45.

Three years have passed in the present case, with no observable relapse. As regional lymph nodes account for 55 % of the first metastatic sites in malignant melanoma cases at the advanced stage [11], the observed long-term survival in this case may be attributable to the lack of lymph node involvement in the resected specimens, effective resection of the primary lesion, and a positive response to the postoperative DAV chemotherapy. DITC was first introduced in Japan in 1977, and tripartite DAV therapy has been used in many institutions. In cases of primary cutaneous malignant melanoma, DTIC alone has an approximate response rate of 20 %, whereas DAV therapy has a reported response rate of 37.5 % [12], although the effect on prolongation of OS of DAV therapy over DTIC alone has not been ascertained [13]. There are currently no reports of the use of this regimen for primary gastrointestinal malignant melanoma. In malignant melanoma of the skin, postoperative DAV-feron therapy (incorporation of interferon-beta with DAV) was reported to possibly improve the OS rate [13]. As this case showed no skin involvement, DAV therapy alone was chosen.

## Conclusions

In conclusion, it is difficult to diagnose primary small intestinal malignant melanoma before surgical resection, and thus, AMM is often detected at advanced stages. The present case of malignant melanoma diagnosed by biopsy of a gastric lesion before surgical resection is rare, and this is the first study to report a diagnosis of AMM before surgery.
